# Multidimensional assessment of fatigue in patients with brain metastases before and after Gamma Knife radiosurgery

**DOI:** 10.1007/s11060-019-03240-w

**Published:** 2019-07-26

**Authors:** Eline Verhaak, Wietske C. M. Schimmel, Margriet M. Sitskoorn, Marjan Bakker, Patrick E. J. Hanssens, Karin Gehring

**Affiliations:** 1 Gamma Knife Center, Elisabeth-TweeSteden Hospital, Tilburg, The Netherlands; 2 Department of Neurosurgery, Elisabeth-TweeSteden Hospital, Tilburg, The Netherlands; 3 Department of Cognitive Neuropsychology, Tilburg University, Tilburg, The Netherlands; 4 Department of Methodology and Statistics, Tilburg University, Tilburg, The Netherlands

**Keywords:** Brain metastases, Cancer, Fatigue, Multidimensional fatigue inventory, Patient reported outcomes, Radiosurgery

## Abstract

**Purpose:**

Fatigue is a common and distressing symptom in cancer patients which negatively affects patients’ daily functioning and health-related quality of life. The aim of this study was to assess multidimensional fatigue in patients with brain metastases (BM) before, and after Gamma Knife radiosurgery (GKRS).

**Methods:**

Patients with BM, an expected survival > 3 months, and a Karnofsky Performance Status ≥ 70 and 104 Dutch non-cancer controls were recruited. The Multidimensional Fatigue Inventory (MFI), measuring general fatigue, physical fatigue, mental fatigue, reduced activity and reduced motivation, was used. Baseline levels of fatigue between patients and controls were compared using independent-samples t-tests. The course of fatigue over time, and clinical and psychological predictors thereof, were analyzed using linear mixed models (within-group analyses).

**Results:**

Ninety-two, 67 and 53 patients completed the MFI at baseline, and 3 and 6 months after GKRS. Before GKRS, patients with BM experienced significantly higher levels of fatigue on all subscales compared to controls (medium to large effect sizes). Over 6 months, general and physical fatigue increased significantly (*p* = .009 and *p* < .001), and levels of mental fatigue decreased significantly (*p* = .027). No significant predictors of the course of fatigue over time could be identified.

**Conclusions:**

Fatigue is a major problem for patients with BM. Different patterns over time were observed for the various aspects of fatigue in patients with BM. Information on the various aspects of fatigue is important because fatigue may negatively affect patients' functional independence, health-related quality of life, and adherence to therapy.

**Electronic supplementary material:**

The online version of this article (10.1007/s11060-019-03240-w) contains supplementary material, which is available to authorized users.

## Introduction

Brain metastases (BM) are the most common type of brain tumors [1]. Life expectancy of patients with BM is increasing mainly due to better systemic treatment of the primary tumor [2, 3]. Fatigue is one of the most distressing symptoms experienced by cancer patients [4, 5] and the most frequently reported symptom in patients with brain tumors in general [6]. Persistent feelings of fatigue can negatively affect a patient’s daily, physical, and social functioning, self-esteem, and health-related quality of life (HRQoL) [4, 7, 8].

Thus far, in one study sample [9, 10] only, levels of fatigue in patients with BM were evaluated before stereotactic radiosurgery (SRS). These patients (n = 97) experienced significantly more fatigue pre-SRS compared to controls and a significant increase of self-reported levels of fatigue over 6 months’ time was noted [9]. In a subsequent publication, on the same study sample, 61% of the patients reported an increase of self-reported fatigue 3 months after SRS [10].

In these two studies, a unidimensional self-report questionnaire was used to measure fatigue. A unidimensional questionnaire measures a patient’s global level of fatigue [11]. More comprehensive are multidimensional questionnaires, including at least two dimensions of fatigue, such as mental fatigue and physical fatigue [5]. Multidimensional assessments better reflect the complex nature of fatigue [5].

Among patients with cancer, levels of fatigue are generally associated with psychological factors such as anxiety and depression, and not with treatment or demographic characteristics [4, 5, 12]. In patients with BM, volume and number of BM did not appear to influence fatigue [9]. Patients with Karnofsky Performance Status (KPS) lower than 90 however had significantly worse levels of fatigue compared to patients with KPS equal to or higher than 90 [9]. In addition, increased levels of fatigue seem to be clustered with a deterioration in global health status [10].

In the current study a multidimensional self-report fatigue questionnaire was used to analyze different aspects of fatigue in patients with (multiple) BM before, and up to 6 months after Gamma Knife radiosurgery (GKRS). As feelings of fatigue are also very common in the general population [5], levels of fatigue between patients with BM were compared to non-cancer controls. Additionally, potential predictors, including both clinical and psychological factors, of the course of the different aspects of fatigue over time were explored.

## Methods

This study is part of a larger prospective longitudinal observational study (CAR-Study A; ClinicalTrials.gov Identifier: NCT02953756) on cognitive functioning over time after GKRS in patients with BM. Secondary outcome measures included patient reported outcomes (anxiety and depression, fatigue, and health-related quality of life). The study was approved by the Medical Ethics Committee Brabant (File NL53472.028.15).

### Patients and procedure

A cohort of adult patients with 1–10 BM representative of daily clinical practice, scheduled for GKRS, was recruited in the Elisabeth-TweeSteden hospital in Tilburg, the Netherlands. Inclusion criteria included: total volume of the BM ≤ 30 cm^3^, KPS ≥ 70 and expected survival > 3 months. Exclusion criteria included: small cell lung cancer, meningeal disease or prior brain radiation/surgery. Full eligibility criteria were described previously [13].

During the first consultation visit, the radiation-oncologist screened for study eligibility, after which eligible patients received detailed information about the study and its procedures. If a patient was willing to participate, a neuropsychological assessment (NPA) was scheduled in the morning before treatment. It took approximately 60 min to complete tests and questionnaires. All patients gave written informed consent.

Patients underwent a contrast-enhanced MRI-scan for treatment planning. Depending upon the volume of the BM, a dose of 18–25 Gy was given with 99–100% coverage of the target. Dose limits for organs at risk are as follows: brainstem 18 Gy and optic chiasm or optic nerves 8 Gy. Before January 2016, patients were treated with the Leksell Gamma Knife^®^ Perfexion™, hereafter with the Leksell Gamma Knife^®^ Icon™. This upgrade did not affect treatment parameters for patients included in this study.

Follow-up assessments took place 3 and 6 months after GKRS combined with the usual care MRI-scans and consultation with the radiation-oncologist. These follow-up MRI-scans were T1-weighted, contrast-enhanced images at 1.5 mm slice thickness. Total volume of the BM was determined at baseline and at 3 and 6 months after GKRS. Partial response was defined as a ≥ 65% decrease in total tumor volume and no new BM. Progressive disease was defined as a ≥ 73% increase in total tumor volume or the appearance of new BM. Stable disease was defined as no partial response or progressive disease. All lesions > 0.523 cm^3^ were considered as targets and were used to evaluate treatment response [14].

Adult Dutch non-cancer controls were recruited by convenience sampling from the broad network of the research group. With this sampling technique, a group of controls could be recruited that had comparable age, proportions of men and women, and educational levels as the patient group (comparable group matching). Inclusion criteria were: no (history of) cancer and, no cerebrovascular disease in the past 12 months. If interested to participate, controls received an information letter. All controls signed informed consent. The same tests and questionnaires as were used in the patient group, and in the same order, were administered at the first assessment, and at 3 and 6 months thereafter. The NPA was, depending on the control’s preference, administered at the control’s home, the university, or the hospital.

### Measures

The Multidimensional Fatigue Inventory (MFI) [15], a 20-item self-report instrument, was used to measure five aspects of fatigue; general fatigue, physical fatigue, mental fatigue, reduced activity and reduced motivation. The responder indicated on a 5-point scale to what extent the statement applied to him or her based on the preceding week (range 4 to 20 points per subscale). A higher raw score indicates more fatigue [15, 16]. In case of any missing items, subscale total scores were not calculated. Multiple linear regression analyses were used to regress raw fatigue scores of the control group on age and sex to generate normative formulae [17]. Raw fatigue scores of the patients were converted into age and sex corrected Z scores using the following formula: Z score = Y_o_ − Y_p_/SD_residual_. Y_o_ is the raw fatigue score of the individual, Y_p_ is the predicted raw fatigue score using regression-based formulae (based on the control group, including age and sex as covariates), and SD_residual_ is the SD (standard deviation) of the control group’s residual (see for example Rijnen et al. [18]). Lower Z scores indicate more severe fatigue. Widely-used cut-offs of 1.3 (90th percentile) and 2.0 (97.5th percentile) were used to determine ‘high’ and ‘very high’ levels of fatigue [19, 20].

Before administration of the MFI, a short neuropsychological test battery including six cognitive tests, and questionnaires concerning anxiety and depression (Hospital Anxiety and Depression Scale; HADS) [21] and HRQoL (Functional Assessment of Cancer Therapy-Brain; FACT-Br) [22] were administered. The HADS is a self-report measure consisting of seven anxiety items and seven depression items, with each item ranging from 0 to 3. Higher scores indicate more symptoms of anxiety or depression within the preceding week [21]. The FACT-Br is a self-report questionnaire consisting of 5 subscales, 2 total scales, and 1 index. One of the total scales, the FACT-General, measures overall HRQoL. Questions are answered on a 5-point Likert scale ranging from 0 (not at all) to 4 (very much). Higher scores indicate a better HRQoL [22-24].

Socio-demographic and clinical characteristics were retrieved from patient’s medical files. Timing of diagnosis of BM within 30 days of the diagnosis of the primary tumor was defined as synchronous, and after 30 days as metachronous.

### Statistical analysis

Statistical analyses were performed with the Statistical Package for the Social Sciences (SPSS) version 24.0 (IBM Corporate Headquarters, Armonk, New York), except for the linear mixed models that were performed in R [25], version 1.1.442. A corrected alpha, by employing the procedure of Benjamini and Hochberg [26], was used to reduce the false discovery rate due to multiple testing per hypothesis.

Characteristics of patients and controls were compared with an independent-samples t-test for age and with a chi-square test of homogeneity for sex and educational level. Kaplan–Meier curves were used to analyse overall survival.

For each aspect of fatigue, the number of patients and controls with a Z score between − 1.3 and − 1.99, and a Z score ≤ − 2.0 were counted to determine the prevalence of patients and controls who experienced ‘high’ and ‘very high’ levels of fatigue, respectively. To compare the proportions of high and very high fatigue between the patients and controls, chi-square tests for homogeneity were performed.

Independent-samples t-tests were conducted to compare baseline mean raw MFI scores between patients and controls. Glass’s delta effect sizes were calculated, by dividing the difference between the means of the groups by the standard deviation of the control group for each MFI scale. An effect size ≤ 0.49 was considered a ‘small’ effect, from 0.50 to 0.79 a ‘medium’ effect and ≥ 0.80 a ‘large’ effect [27].

The *nlme* package [28] in R [25] was used to perform a set of linear mixed models of the relationship between scores on each fatigue scale and time within the group of patients with BM. To estimate the model parameters, the restricted maximum likelihood estimate (REML) method was used. The Akaike Information Criterion (AIC) and Bayesian Information Criterion (BIC) were used to estimate model fit. Intercepts for subjects for the effect of fatigue were added as random intercepts, this ensured that data over time were estimated individually for each patient before a general trend was estimated. Random slopes were not added as they did not improve model fit [29]. The best fit was provided by a first-order autoregressive covariance structure (AR1) at level 1 and a Scaled Identity matrix at level 2. Available data of patients with some missing data were used (e.g. data at baseline and 3 months was still used if there was no data at 6 months).

Additionally, time was added as categorical variable, with 3 months as reference category, to examine differences in fatigue between baseline and 3 months and between 3 and 6 months.

Lastly, 5 pre-GKRS predictors of the course of fatigue over time, based on findings in previous studies [4, 5, 9, 10, 12], were entered into the model as fixed factors at the individual level. These variables were clinical (KPS and timing of diagnosis of BM), or psychological (general HRQoL, symptoms of anxiety and symptoms of depression).

## Results

### Sociodemographic characteristics

Characteristics of 92 patients with BM and 104 controls are presented in Table 1. The patient and control group did not differ significantly regarding age, sex and education.

**Table 1:** Characteristics

	No. of patients (%)	No. of controls (%)	*t* score	*χ* ^2^	p*
Number of participants	92	104			
Age in years, median (range)	63 (31–80)	60 (31–87)	1.527		.128
Sex, male	47 (51.1)	50 (48.1)		0.177	.674
Education^a^					
Low	28 (30.4)	25 (24.0)		4.626	.099
Middle	37 (40.2)	33 (31.7)			
High	27 (29.3)	46 (44.2)			
Histology of the primary cancer					
Lung	55 (59.8)				
Renal	15 (16.3)				
Melanoma	12 (13.0)				
Breast	6 (6.5)				
Other	4 (4.3)				
Systemic treatment before or at GKRS					
No	39 (42.4)				
Yes	53 (57.6)				
Chemotherapy	17 (18.5)				
Chemo-radiotherapy	11 (12.0)				
Targeted therapy	11 (12.0)				
Chemo- and immunotherapy	4 (4.3)				
Chemo- and targeted therapy	3 (3.3)				
Chemo- and hormonal therapy	2 (2.2)				
Immuno- and targeted therapy	2 (2.2)				
Immunotherapy	1 (1.1)				
Chemo-, immuno-, and hormonal therapy	1 (1.1)				
Chemo-, immuno-, hormonal, and targeted therapy	1 (1.1)				
Use of dexamethasone at GKRS					
No	29 (31.5)				
Yes	63 (68.5)				
KPS, median (range)	90 (70–100)				
RPA					
Class 1	16 (17.4)				
Class 2	76 (82.6)				
GPA					
Class 2	15 (16.3)				
Class 3	60 (65.2)				
Class 4	17 (18.5)				
Number of brain metastases					
1	32 (34.8)				
2–4	29 (31.5)				
5–10	31 (33.7)				
Total tumor volume, cm^3^, median (range)^b^	5.6 (0.02–31.1)				

*No.* number, *KPS* Karnofsky Performance Status, *RPA* recursive partitioning analysis, *GPA* graded prognostic assessment, *BM* brain metastases

*Corrected alpha of .02, using the Benjamini–Hochberg procedure [25]

^a^The seven categories to classify the level of education of the Verhage scale [37] were merged into low (Verhage 1–4), middle (Verhage 5), and high (Verhage 6 and 7) educational level

^b^One patient had a total tumor volume 31.1 cm^3^ on the MRI-scan used for treatment planning

## Compliance and survival

At baseline, 92 patients (100%) and 102/104 (98%) controls completed the MFI. At 3 months, 67 of 76 patients alive (88%) and at 6 months, 53 of 65 patients alive (82%) completed the MFI. The median overall survival was 11.8 months (95% CI, 8.6 to 15.0 months; at time of analysis 65 patients (70.7%) had died). The 1-year survival rate was 48.9%. Of 67 evaluable patients (patients with at least 1 follow-up assessment), 26 patients (38.8%) had intracranial progression (solely due to the appearance of new lesions in 15 patients (57.7%)), one patient had pseudo-progression (1.5%), 18 patients (26.9%) had a partial response and no new BM, and 22 patients (32.8%) had stable disease.

## Pre-GKRS fatigue

At group level, patients with BM experienced significantly higher levels of fatigue on all subscales compared to the control group (*p* ≤ .001), with the highest effect sizes for reduced activity and mental fatigue (Table 2). On the individual level, significantly higher proportions of patients experienced high and very high fatigue on at least one of five subscales compared to controls (53.3% vs. 26.0%, χ^2^ = 14.883, *p* =  < .001, and 35.6% vs. 15.0%, χ^2^ = 10.750, *p* = .001, respectively). In addition, significantly more patients, compared to controls, experienced high fatigue on the scales physical fatigue and reduced activity, and very high fatigue on general fatigue, mental fatigue, reduced activity and reduced motivation (Table 2).

**Table 2:** Group and individual pre-GKRS fatigue scores (MFI) of patients and controls

Group-level	Patients with BM (n = 92)		Control group (n = 102)		Patients with BM vs. non-cancer controls					
	Mean	SD	Mean	SD	*t*-score	p*	Mean diff	95% Confidence interval		Effect size^d^
								Lower	Upper	
General fatigue^a^	11.5	4.3	8.8	3.8	4.725	**<** **.001**	2.76	1.609	3.914	0.71
Physical fatigue	10.7	4.6	8.6	4.2	3.366	**.001**	2.14	0.886	3.392	0.50
Mental fatigue^b^	11.3	4.0	8.2	3.7	5.476	**<** **.001**	3.04	1.944	4.133	0.84
Reduced activity^a^	11.7	4.0	8.3	3.4	6.486	**<** **.001**	3.47	2.415	4.526	1.00
Reduced motivation^b,c^	9.3	3.8	7.4	3.1	3.669	**<** **.001**	1.84	0.852	2.835	0.61

Individual-level	Percentages of high fatigue (− 1.3 > Z > − 2.0)		Percentages of very high fatigue (Z ≤ − 2.0)		Patients with BM vs. control group			
					High fatigue		Very high fatigue	
	Patients (n = 92) (%)	Controls (n = 102) (%)	Patients (n = 92) (%)	Controls (n = 102) (%)	χ^2^	p**	χ^2^	p**
General fatigue^a^	19.6	11.9	13.0	3.0	2.165	.141	6.815	**.009**
Physical fatigue	18.5	6.9	12.0	5.9	6.020	**.014**	2.232	.135
Mental fatigue^b^	18.7	8.8	14.3	2.0	4.009	.045	10.192	**.001**
Reduced activity^a^	26.1	5.9	19.6	5.9	14.885	**<** **.001**	8.208	**.004**
Reduced motivation^b^	19.8	10.8	16.5	4.9	3.048	.081	6.945	**.008**

*MFI* multidimensional fatigue inventory, *BM* brain metastases, *n* number of participants, *SD* standard deviation, *mean diff* mean difference

*Corrected alpha of .05, **corrected alpha of .03, using the Benjamini–Hochberg procedure [25]

^a^Number of controls = 101

^b^Number of patients with BM = 91

^c^Equal variances not assumed

^d^Glass’s delta

## Fatigue over time

Over 6 months, patients’ mean levels of general and physical fatigue increased significantly and patients’ levels of mental fatigue decreased significantly (Table 3). There was no significant change in levels of reduced activity and reduced motivation. Between pretreatment and 3 months, there was a significant increase in levels of general and physical fatigue, and these levels of fatigue remained stable between 3 and 6 months. For levels of mental fatigue there was a significant decrease over 6 months, but there was no significant change between baseline and 3 months nor between 3 and 6 months after GKRS.

**Table 3:** Fatigue scores of patients with BM over time

Fatigue subscales	Control group Mean (SD) (n = 102)	Patients with BM Mean (SD) per month			Time slope beta (SE)	*F* value	p*	Baseline vs. 3 months		3 Months vs. 6 months	
		0 (n = 92)	3 (n = 67)	6 (n = 53)				Beta (SE)	p**	Beta (SE)	p**
General fatigue	8.8 (3.8)^a^	11.5 (4.3)	13.2 (4.5)	13.1 (4.6)	0.8 (.3)	7.125	**.009**	1.7 (.5)	**0.002**	− 0.3 (.5)	.569
Physical fatigue	8.6 (4.2)	10.7 (4.6)	13.1 (4.8)	13.2 (4.9)	1.2 (.3)	18.040	**<.001**	2.3 (.5)	**<0.001**	− 0.1 (.5)	.878
Mental fatigue	8.2 (3.7)	11.3 (4.0)^b^	10.4 (4.5)	10.3 (4.3)	− 0.6 (.3)	5.001	**.027**	− 0.8 (.5)	0.112	− 0.3 (.5)	.494
Reduced activity	8.3 (3.4)^a^	11.7 (4.0)	12.4 (4.6)	11.9 (4.2)	0.1 (.3)	0.028	.867	0.6 (.6)	0.279	− 0.6 (.5)	.175
Reduced motivation	7.4 (3.1)	9.3 (3.8)^b^	10.1 (4.0)	10.0 (3.6)	0.4 (.3)	1.983	.162	0.9 (.5)	0.075	− 0.3 (.4)	.546

*BM* brain metastases, *n* number of patients, *SD* standard deviation, *SE* standard error, *vs.* versus

*Corrected alpha of .03; **corrected alpha of .01, using the Benjamini–Hochberg procedure [25]

^a^Number of controls = 101

^b^Number of patients with BM = 91

## Predictors of the course of fatigue

None of the predefined clinical and psychological factors significantly predicted the different aspects of fatigue over time (table presented in Online Resource 1).

## Discussion

Our findings suggest that fatigue is not only a rather common, but also a severe problem in patients with BM. Pretreatment group level results showed that patients with BM experienced significantly higher levels of fatigue on all five aspects compared to the controls, with medium to large effect sizes. At the individual level, respectively 53.3% and 35.6% of the patients experienced high or very high fatigue on at least one aspect of fatigue pre-GKRS. For each separate fatigue scale significantly higher proportions of patients experienced high or very high fatigue compared to the controls.

Different patterns over time were observed for the various aspects of fatigue in patients with BM. Over 6 months, general and physical fatigue significantly increased, whereas mental fatigue significantly decreased. Reduced activity and reduced motivation did not change significantly during this period. This emphasizes the importance of multidimensional fatigue measures, as a unidimensional score would not have been able to capture these different fatigue patterns and changes over time. Further analyses on the specific time intervals indicated that levels of general and physical fatigue increased significantly during the first 3 months after GKRS and did not change between 3 and 6 months after GKRS. In line with this, Habets et al. [9], reported an increase in levels of fatigue over 6 months, and in the subsequent publication of van der Meer et al. [10] in the same study sample, most patients experienced increased levels of fatigue 3 months after SRS, and stable levels of fatigue 6 months after SRS. However, in both studies only global fatigue was assessed as fatigue was measured with a unidimensional questionnaire.

The higher baseline levels of fatigue of patients with BM could be caused by the effects of tumor burden. Tumor burden can lead to inflammation, changes in cytokine levels, and dysregulations of the hypothalamic–pituitary–adrenal axis, which in turn may lead to reduced energy, fatigue, anemia and malaise [30-33]. Worsened general and physical fatigue 3 months after GRKS could be due to radiation-induced fatigue, which is a common early side effect of radiation [34]. Mental distractions and preoccupations due to being diagnosed with a life-threatening disease and the upcoming treatment with GKRS might lead to increased mental fatigue pre-GKRS. As patients get more adapted to being diagnosed with BM over time, these mental distractions may gradually decline resulting in a decrease of mental fatigue.

In line with the few previous studies evaluating predictors of fatigue in patients with cancer [4, 5, 12], none of the pre-SRS clinical predictors (KPS and synchronous vs. metachronous diagnosis of BM) predicted the course of fatigue over time. In a previous study in patients with BM [9], KPS was a predictor for fatigue in univariate analyses whereas in the current study, using multivariable analyses, KPS was not a significant predictor. Unlike previous studies [4, 5, 10, 12], none of our pre-GKRS psychological factors (general HRQoL, anxiety, and depression) predicted levels of fatigue over time. In a sample of patients with breast cancer, style and intensity of coping were related to experiences of fatigue [35], whereby passive coping strategies were related to persistent fatigue [36]. Patients’ personality traits were more strongly related to fatigue than demographic, treatment or disease-related factors [37]. Since multiple studies indicated that treatment or disease-related factors are not predictive for the course of fatigue over time [5, 12], more patient-specific factors, such as personality or coping style, should be investigated in future studies including patients with BM. However, specific factors for this patient group, such as different primary tumors, differences in systemic treatments (e.g., chemotherapy, immunotherapy, combined modality therapy), neurologic disease burden, and use of steroids, may still play a role in levels of fatigue over time.

There are several behavioral interventions that may diminish fatigue in patients with central nervous system (CNS) and non-CNS cancers, including coping strategies, education, exercise, rest and sleep, energy conservation, stress reduction, and cognitive rehabilitation [4, 38-40]. Future studies should evaluate if these interventions are also feasible and effective for patients with BM. As feelings of general and physical fatigue increase in the acute phase after GKRS and remain present over time, patients will most likely benefit from interventions addressing both of these aspects.

Although a heterogeneous study sample, consisting of patients with several types of primary cancers, was included, it is representative for daily clinical practice. Participation in the current study may have been burdensome for patients as they had to complete several neuropsychological tests in addition to filling out the questionnaire concerning fatigue. As a consequence, patients who participated in this study, might have been more resilient and perhaps less fatigued than non-participating patients. On the other hand, the fatigue questionnaire was completed in the morning before treatment and at clinical follow-ups (including MRI-scan and consult), during which, patients may have experienced additional anxiety or fatigue.

Feelings of fatigue occur significantly more frequently in patients with BM before GKRS than in non-cancer controls. Excessive feelings of fatigue on all different aspects were present during at least 6 months, which may negatively affect a patients’ socio-professional functioning, independence, HRQoL, mood, cognitive functioning and adherence to therapy [7, 41, 42]. Another consequence of being highly fatigued may be that patients withdraw themselves from social and/or family life (e.g., being too fatigued to engage in, or enjoy, social interactions with family members, friends, and colleagues).

The various aspects of fatigue showed different pretreatment levels and patterns over time, indicating the importance of incorporating a multidimensional fatigue measure in clinical practice. In order to provide patients with adequate information and recommendations on interventions for fatigue, awareness among health-care professionals of this disruptive symptom should be increased. The results further indicate that it is of interest to invest in research on therapies targeting fatigue.

## Electronic supplementary material

Below is the link to the electronic supplementary material.

Supplementary file1 (DOCX 19 kb)

## References

[1] Kaal EC, Niël CG, Vecht CJ (2005). Therapeutic management of brain metastasis. Lancet Neurol.

[2] Jensen CA, Chan MD, McCoy TP, Bourland JD, DeGuzman AF, Ellis TL (2011). Cavity-directed radiosurgery as adjuvant therapy after resection of a brain metastasis. J Neurosurg.

[3] Nayak L, Lee EQ, Wen PY (2012). Epidemiology of brain metastases. Curr Oncol Rep.

[4] Ahlberg K, Ekman T, Gaston-Johansson F, Mock V (2003). Assessment and management of cancer-related fatigue in adults. Lancet.

[5] Stone PC, Minton O (2008). Cancer-related fatigue. Eur J Cancer.

[6] Noh T, Walbert T, Noh T, Walbert T (2018). Brain metastasis: clinical manifestations, symptom management, and palliative care. Handbook of clinical neurology.

[7] Curt GA, Breitbart W, Cella D, Groopman JE, Horning SJ, Itri LM (2000). Impact of cancer-related fatigue on the lives of patients: new findings from the Fatigue Coalition. Oncologist.

[8] Magnusson K, Möller A, Ekman T, Wallgren A (1999). A qualitative study to explore the experience of fatigue in cancer patients. Eur J Cancer Care.

[9] Habets EJJ, Dirven L, Wiggenraad RG, Verbeek-de Kanter A, Lycklama A, Nijeholt GJ, Zwinkels H (2016). Neurocognitive functioning and health-related quality of life in patients treated with stereotactic radiotherapy for brain metastases: a prospective study. Neuro-Oncology.

[10] van der Meer PB, Habets EJ, Wiggenraad RG, Verbeek-de Kanter A, Nijeholt GJL, Zwinkels H (2018). Individual changes in neurocognitive functioning and health-related quality of life in patients with brain oligometastases treated with stereotactic radiotherapy. J Neurooncol.

[11] Jacobsen PB (2004). Assessment of fatigue in cancer patients. JNCI Monogr.

[12] Prue G, Rankin J, Allen J, Gracey J, Cramp F (2006). Cancer-related fatigue: a critical appraisal. Eur J Cancer.

[13] Verhaak E, Gehring K, Hanssens PEJ, Sitskoorn MM (2019). Health-related quality of life of patients with brain metastases selected for stereotactic radiosurgery. J Neurooncol.

[14] Lin NU, Lee EQ, Aoyama H, Barani IJ, Barboriak DP, Baumert BG (2015). Response assessment criteria for brain metastases: proposal from the RANO group. Lancet Oncol.

[15] Smets E, Garssen B, Bonke BD, De Haes J (1995). The Multidimensional Fatigue Inventory (MFI) psychometric qualities of an instrument to assess fatigue. J Psychosom Res.

[16] Smets E, Garssen B, Cull A, De Haes J (1996). Application of the multidimensional fatigue inventory (MFI-20) in cancer patients receiving radiotherapy. Br J Cancer.

[17] Oosterhuis HE, van der Ark LA, Sijtsma K (2016). Sample size requirements for traditional and regression-based norms. Assessment.

[18] Rijnen SJ, Meskal I, Emons WH, Campman CA, van der Linden SD, Gehring K (2017). Evaluation of normative data of a widely used computerized neuropsychological battery: applicability and effects of sociodemographic variables in a Dutch sample. Assessment.

[19] Lezak M, Howieson D, Loring D (2012). Neuropsychological assessment.

[20] Bouma A, Mulder J, Lindeboom J, Schmand B (2012). Handboek neuropsychologische diagnostiek.

[21] Zigmond AS, Snaith RP (1983). The hospital anxiety and depression scale. Acta Psychiatr Scand.

[22] FACIT.org (2017). FACIT: providing a voice for patients worldwide. https://www.facit.org.

[23] Cella DF, Tulsky DS, Gray G, Sarafian B, Linn E, Bonomi A (1993). The Functional Assessment of Cancer Therapy scale: development and validation of the general measure. J Clin Oncol.

[24] Weitzner MA, Meyers CA, Gelke CK, Byrne KS, Levin VA, Cella DF (1995). The functional assessment of cancer therapy (FACT) scale Development of a brain subscale and revalidation of the general version (FACT-G) in patients with primary brain tumors. Cancer.

[25] R Core Team (2017). R: a language and environment for statistical computing.

[26] Benjamini Y, Hochberg Y (1995). Controlling the false discovery rate: a practical and powerful approach to multiple testing. J R Stat Soc B.

[27] Cohen J (1988). Statistical power analysis for the behavioral sciences.

[28] Pinheiro J, Bates D, DebRoy S, Sarkar D, R Core Team (2018) Linear and nonlinear mixed effects models. R package, 3.1-137

[29] West BT, Welch KB, Galecki AT (2014). Linear mixed models: a practical guide using statistical software.

[30] Shi C, Lamba N, Zheng L, Cote D, Regestein Q, Liu C (2018). Depression and survival of glioma patients: a systematic review and meta-analysis. Clin Neurol Neurosurg.

[31] Spiegel D, Giese-Davis J (2003). Depression and cancer: mechanisms and disease progression. Biol Psychiat.

[32] Strain JJ, Blumenfield M (2018). Depression as a systemic illness.

[33] Kurzrock R (2001). The role of cytokines in cancer-related fatigue. Cancer.

[34] Jereczek-Fossa BA, Marsiglia HR, Orecchia R (2002). Radiotherapy-related fatigue. Crit Rev Oncol Hematol.

[35] Reddick BK, Nanda JP, Campbell L, Ryman DG, Gaston-Johansson F (2006). Examining the influence of coping with pain on depression, anxiety, and fatigue among women with breast cancer. J Psychosoc Oncol.

[36] Gélinas C, Fillion L (2004). Factors related to persistent fatigue following completion of breast cancer treatment. Oncol Nurs Forum.

[37] Michielsen HJ, Van der Steeg AF, Roukema JA, De Vries J (2007). Personality and fatigue in patients with benign or malignant breast disease. Support Care Cancer.

[38] Gehring K, Sitskoorn MM, Gundy CM, Sikkes S, Klein M, Postma TJ (2009). Cognitive rehabilitation in patients with gliomas: a randomized, controlled trial. J Clin Oncol.

[39] Gehring K, Kloek CJ, Aaronson NK, Janssen KW, Jones LW, Sitskoorn MM (2018). Feasibility of a home-based exercise intervention with remote guidance for patients with stable grade II and III gliomas: a pilot randomized controlled trial. Clin Rehabil.

[40] Day J, Yust-Katz S, Cachia D, Wefel J, Katz LH, Tremont Lukats IW (2016). Interventions for the management of fatigue in adults with a primary brain tumour. Cochrane Libr Syst Rev.

[41] Bower JE, Lamkin DM (2013). Inflammation and cancer-related fatigue: mechanisms, contributing factors, and treatment implications. Brain Behav Immun.

[42] Bower JE, Ganz PA, Desmond KA, Rowland JH, Meyerowitz BE, Belin TR (2000). Fatigue in breast cancer survivors: occurrence, correlates, and impact on quality of life. J Clin Oncol.

