# Supplementary material for: Multidimensional assessment of fatigue in patients with brain metastases before and after Gamma Knife radiosurgery
Source: J Neurooncol. 2019 Jul 26;144(2):377–84. doi: 10.1007/s11060-019-03240-w (PMC6700236; doi:10.1007/s11060-019-03240-w)
Supplement: Supplementary file 1 — Supplementary file1 (DOCX 19 kb) [file 11060_2019_3240_MOESM1_ESM.docx]

**Journal of Neuro-Oncology – Online Resource 1**

**Multidimensional assessment of fatigue in patients with brain metastases before and after Gamma Knife radiosurgery**

**Eline Verhaak^1,2,3^, Wietske C.M. Schimmel^1,2,3^, Margriet M. Sitskoorn^2,3^, Marjan Bakker^4^, Patrick E. J. Hanssens^1,2^, AND Karin Gehring^1,2,3^**

^1^ Gamma Knife Center, Elisabeth-TweeSteden Hospital, Tilburg, The Netherlands

^2^ Department of Neurosurgery, Elisabeth-TweeSteden Hospital, Tilburg, The Netherlands

^3^ Department of Cognitive Neuropsychology, Tilburg University, Tilburg, The Netherlands

^4^ Department of Methodology and Statistics, Tilburg University, Tilburg, The Netherlands

**Corresponding author:** Karin Gehring (E-mail: k.gehring@tilburguniversity.edu, Telephone number: +31 13 466 4233)

**Predictors of the course of fatigue over time – CAR-Study A** (NCT02953756)

| **Supplementary Table 1.** Predictors of levels of fatigue over 6 months’ time after Gamma Knife radiosurgery of patients with BM | | | | | |
| --- | --- | --- | --- | --- | --- |
| Domain of fatigue | Predictor | *b* | *SE* | F | *p^*^* |
| General Fatigue | General HRQoL | 0.054 | 0.03 | 4.268 | .041 |
|  | Anxiety | -0.168 | 0.09 | 3.786 | .054 |
|  | Depression | 0.214 | 0.11 | 3.764 | .055 |
|  | KPS | -0.007 | 0.03 | 0.039 | .843 |
|  | Synchronous (ref) versus metachronous diagnosis of BM | 1.072 | 0.63 | 2.864 | .093 |
| Physical Fatigue | General HRQoL | 0.053 | 0.03 | 3.997 | .048 |
|  | Anxiety | -0.154 | 0.09 | 3.114 | .080 |
|  | Depression | 0.178 | 0.11 | 2.552 | .113 |
|  | KPS | 0.003 | 0.03 | 0.009 | .924 |
|  | Synchronous (ref) versus metachronous diagnosis of BM | 0.930 | 0.64 | 2.131 | .147 |
| Mental Fatigue | General HRQoL | -0.052 | 0.02 | 4.901 | .029 |
|  | Anxiety | -0.087 | 0.08 | 1.240 | .268 |
|  | Depression | -0.133 | 0.10 | 1.757 | .188 |
|  | KPS | 0.002 | 0.03 | 0.006 | .938 |
|  | Synchronous (ref) versus metachronous diagnosis of BM | 0.982 | 0.57 | 2.958 | .088 |
| Reduced Activity | General HRQoL | 0.014 | 0.03 | 0.256 | .614 |
|  | Anxiety | -0.132 | 0.10 | 1.925 | .168 |
|  | Depression | 0.057 | 0.12 | 0.227 | .635 |
|  | KPS | -0.007 | 0.04 | 0.030 | .863 |
|  | Synchronous (ref) versus metachronous diagnosis of BM | 0.617 | 0.69 | 0.793 | .375 |
| Reduced Motivation | General HRQoL | 0.009 | 0.03 | 0.129 | .720 |
|  | Anxiety | -0.014 | 0.09 | 0.025 | .875 |
|  | Depression | -0.064 | 0.11 | 0.339 | .561 |
|  | KPS | -0.005 | 0.03 | 0.024 | .878 |
|  | Synchronous (ref) versus metachronous diagnosis of BM | 0.173 | 0.63 | 0.074 | .786 |
| *Note:* BM: brain metastases, HRQoL: Health-Related Quality of Life, KPS: Karnofsky Performance Status, ref: reference category  ^*^ Corrected alpha’s for each separate LMM, following the Benjamini-Hochberg procedure [1] were used. The corrected alpha was .01 for all MFI scales. | | | | | |

**References**

1. Benjamini, Y., & Hochberg, Y. (1995). Controlling the false discovery rate: a practical and powerful approach to multiple testing. *Journal of the royal statistical society. Series B (Methodological)*, 289-300.
